# Poisoning by the Arsenite of Copper

**Published:** 1847-10

**Authors:** 


*Poisoning by the Arsenite of Copper.*—A child of Mr. Andrew Howe, of Townsend, Mass., was poisoned recently with a green card, which was given to it for a plaything. The fluids of the child's mouth dissolved the green pigment, which it swallowed, producing the most alarming symptoms.

Antidotes and remedies were promptly and perseveringly applied by the attending physician, Dr. Hitchcock, of Ashby. The child is now well. Dr. Hitchcock ascertained that the green paint which ornamented the card, was composed of Scheele's green (arsenite of copper) and carbonate of lead, two most virulent poisons.—*Ibid.*

